# Editorial: New biomarkers for the diagnosis and treatment of systemic lupus erythematosus

**DOI:** 10.3389/fimmu.2022.1009038

**Published:** 2022-10-12

**Authors:** Andras Perl, Nancy Agmon-Levin, José C. Crispín, Trine N. Jorgensen

**Affiliations:** ^1^ Department of Medicine, College of Medicine, State University of New York, Upstate Medical University, Syracuse, NY, United States; ^2^ Department of Biochemistry and Molecular Biology, College of Medicine, State University of New York, Upstate Medical University, Syracuse, NY, United States; ^3^ Department of Microbiology and Immunology, College of Medicine, State University of New York, Upstate Medical University, Syracuse, NY, United States; ^4^ The Zabludowicz Center for Autoimmune Diseases, Sheba Medical Center, Ramat Gan, Israel; ^5^ Department of Immunology and Rheumatology, Instituto Nacional de Ciencias Médicas y Nutrición Salvador Zubirán, Mexico City, Mexico; ^6^ Tecnologico de Monterrey, Escuela de Medicina y Ciencias de la Salud, Monterrey, Mexico; ^7^ Department of Inflammation & Immunity, Lerner Research Institute, Cleveland Clinic, Cleveland, OH, United States

**Keywords:** lupus (SLE), biomarker, multi-omics analyses, machine learning, systems biology

Systemic lupus erythematosus is an autoimmune disease of unknown etiology that primarily affects females of child-bearing age with various morbidities (1). Mortality of SLE still exceeds 10% over 5 years (2, 3). While current treatments are partially effective, they carry significant side effects (4), with infections due to toxicity of immunosuppressant medications being a major cause of death (5, 6). This includes belimumab, the 1^st^ drug approved by the FDA for SLE treatment in 56 years (7), and more recently anifrolumab, both of which also predispose to infections (8). Therefore, a significant unmet need exists to identify biomarkers that can be targeted for safe and effective therapeutic intervention in SLE. A Research Topic centered around new biomarkers for the Diagnosis and Treatment of SLE included 15 publications with a wide range of focus and experimental design. This Editorial addresses the challenges of integrating a series of newly reported single biomarkers, composite biomarkers based multi-omics approaches, and biomarkers based on machine learning with the complex systems biology of SLE. These newly reported biomarkers are shown in **Table 1**.

**Table 1:** New biomarkers for the diagnosis and treatment of SLE.

Biomarker	Source	Outcome	Impact	Reference
*Bacilli*, *Lactobacillales*	Gut	SLE	Risk	(9)
*Bacillales*, *Coprobacter*, *Lachnospira*	Gut	SLE	Protection	(9)
IL-6	Hippocampus	NPSLE	Diagnosis	(10)
Lactoceramide	Plasma	CVD	Diagnosis	(11)
Anti-DNA	Serum	LN	Flare	(12)
ABCB1, IFI27, PLSCR1	PBMC	SLE	Diagnosis	(13)
P3H1, PHACTR4, RGS12	Serum	LN	Diagnosis	(14)
ALCAM, VCAM-1 and PF4	Urine	LN	Flare	(15)
S100A8	Kidney	SLEDAI, LN	Diagnosis	(16)
S100A8	Blood, Urine, Saliva	SLEDAI, LN	Flare	(17)
Adiponectin, MCP-1, sVCAM-1,PF4	Urine	LN	Flare	(18)
CD11c, T-bet, and CD21high B cells	Blood	LN	Protection	(19)

LN, lupus nephritis.

S100 calcium-binding protein A8 protein (S100A8) levels as biomarkers for systemic lupus erythematosus (SLE) were quantified in serum, urine, and saliva samples from 249 patients with SLE and 52 age- and sex-matched healthy controls (HCs) and a receiver operating characteristic curve was used to analyze whether they may be used as biomarkers for diagnosis and prediction of flares (17). For SLE diagnosis, the area under the receiver operating characteristic curve (AUC) was 0.831 for serum S100A8 (95% CI, 0.765–0.897), 0.751 for urine S100A8 (95% CI, 0.648–0.854), and 0.729 for salivary S100A8 (95% CI, 0.646–0.812). Pearson’s correlation analysis showed that S100A8 in serum, urine, and saliva was significantly associated with the SLEDAI (r = 0.267, p < 0.001; r = 0.274, p < 0.001; and r = 0.629, p < 0.001, respectively). Among the clinical manifestations, nephritis was the only organ involvement that was associated with increased concentration of S100A8 in serum, urine, and saliva in comparison to SLE patients without LN (17). An independent study demonstrated that enhanced glomerular S100A8 staining in class IV LN patients over controls (16). S100A8 has been identified as a differentially expressed gene (DEG) with overexpression in kidneys of LN patients (16).

Mass spectroscopy of circulating immune complexes identified 300 proteins in the serum of SLE patients, several of which were found to be highly associated with LN in two independent patient-control cohorts (14). Prolyl 3-hydroxylase 1 (P3H1), phosphatase and actin regulator 4 (PHACTR4), and regulator of G-protein signaling 12 (RGS12) discriminated LN AUC values of 0.82, 0.99, and 0.90, respectively.

Serial kidney biopsies for initial diagnosis and subsequent monitoring of lupus nephritis (LN) remain challenging, thus non-invasive biomarkers are needed. Urinary ALCAM, PF4, and VCAM-1 were identified as potential biomarkers for predicting kidney disease activity in childhood-onset SLE with ALCAM (AUC 0.83) being the single most predictive (15). Herpes virus entry mediator (HVEM) demonstrated comparable diagnostic ability to creatinine normalization when distinguishing active lupus nephritis from inactive SLE patients using the candidate biomarker ALCAM (20). In a 3-stage study including a total of 321 LN patients, a combination of four biomarkers, adiponectin, MCP-1, sVCAM-1 and PF4, were found to have the greatest predictive value for the detection of proliferative, active LN (18). Patients with LN exhibit a profound depletion of atypical age-associated B-cell (ABC) like CD11c^+^T-bet^+^CD21^hi^ B cells in comparison with healthy individuals and SLE patients without LN (19). Selected from 284 DEGs identified in two independent SLE cohorts in the Gene Expression Omnibus (GEO) database, machine learning validated ABCB1, IFI27, and PLSCR1 as top predictors of SLE in a Chinese validation cohort of patients over ethnically matched controls (13). Expression of each these genes was correlated with the expansion of pro-inflammatory lineages of the adaptive and innate immune systems (13). Separately, a comprehensive analysis of gut microbiome genome databases newly identified *Bacilli* and *Lactobacillales* as promoters of SLE and *Bacillales*, *Coprobacter* and *Lachnospira* as protectors from SLE (9).

Among plasma sphingolipids, lactoceramide has been identified as a potential predictor of cardiovascular disease (CVD) in African-American patients with SLE (11). Tan et al. provide an extensive review of biomarker development for SLE (21). The biomarkers are divided by their molecular nature: i) autoantibodies; ii) serum proteins (cytokines, chemokines, complement components, soluble receptors and transporters); iii) microRNAs and long non-coding RNA (LncRNAs); and relevance for organ involvement, such as nephritis, neuropsychiatric lupus, and cutaneous lupus. The review does not discuss cellular biomarkers, such as Tregs, memory B or T cells, or metabolites. Indeed, a comprehensive review of all biomarkers implicated in lupus pathogenesis and patients care remains daunting. In contrast, Ole Petter Rekvig focuses on the role of DNA structure in triggering anti-DNA antibodies and its relationship to lupus nephritis (12). Mitochondrial N-formyl methionine (fMet) is newly implicated in promoting neutrophil-mediated inflammation in systemic sclerosis (22).

Neuropsychiatric SLE (NPSLE) can be diagnosed in the majority of patients with appropriate screening instruments (23). NPSLE and particularly depression has been associated with elevated levels of type 1 interferons, TNFs, and IL-6 in the cerebrospinal fluid (CSF) of SLE patients (24). Accumulation of senescent cells in the hippocampus has been linked to major depression (25). Apparently, depression in lupus-prone MRL/lpr mice is associated with the accumulation of senescent cells in the cornu ammonis 3 (CA3) region of hippocampus (10). Importantly, oral fisetin, a senolytic drug, reduced the number of senescent neural cells and IL-6 mRNA in the hippocampus and improved depressive behavior in the MRL/lpr mice (10).

Given the diversity of hypotheses, methodologies, experimental models and study design, integration of the newly reported biomarkers with the systems biology of SLE present multiple challenges. Such challenges can be easily attributed to a general lack of understanding of lupus pathogenesis. However, several key facts need to be considered when integrating such interesting but diverse outcome. A hallmark of SLE is the production of antinuclear autoantibodies (ANA) (26). Although ANAs are directed to an ever growing number of nucleic acid and nucleoprotein targets and their titers can greatly vary due to the course of disease and impact of therapies, their detection remains a key criterion of diagnostic workup (27). Thus, biomarkers may be mechanistically connected, either upstream or downstream, to the generation or handling of autoantibodies and cell-mediated autoreactivity. T (28) and B cells of the adaptive immune system (29, 30) and IFN-producing dendritic cells are essential for lupus pathogenesis (31). Therefore, integrating biomarkers into the signaling networks that connect the adaptive and innate arms of a dysfunctional immune system is critical for appreciating their significance for controlling pathogenesis, predicting flares or serving as target for treatment in SLE. Notably, only few of these studies involved cellular biomarkers (13, 19) that can be connected to central pathways of lupus pathogenesis. Nevertheless, certain easily detectable biomarkers may also serve other purposes, such as sensing organ damage and obviating the need for invasive procedures, i.e., renal biopsy in LN (15, 17–19). Fisetin was found to control depression by preventing the senescence of neuronal cells in the hippocampus of MRl/lpr mice (10). Fisetin is known to exert its antiaging effect by blocking the mechanistic target of rapamycin (mTOR) (32, 33) (**Figure 1**), which serves a sensor of cellular stress and central regulator of pro-inflammatory lineage development in the immune system (36). Importantly, T cells of SLE patients (37–41) and mice exhibit activation of the mechanistic target of rapamycin (mTOR) (42, 43). Th17 and IL-4 and IL-17-producing DN T cells are expanded, while CD8 EMT cells (44, 45) and Tregs are deficient in SLE patients due to cell type-specific skewing of autophagy that can be corrected with therapeutic efficacy by mTOR blockade (45, 46). Rapamycin blocks nephritis in SLE (47–49). Rapamycin also blocks the production of vascular cell adhesion molecule-1 (VCAM-1) by vascular endothelial cells (50). Of note, VCAM1 was identified as a sensitive biomarker of active LN by two independent studies published under this Research Topic (15, 18). Therefore, it’s possible that mTOR blockade with sirolimus or fisetin would block LN *via* reducing the expression and urinary excretion of VCAM1 and other adhesion molecules.

**Figure 1:**
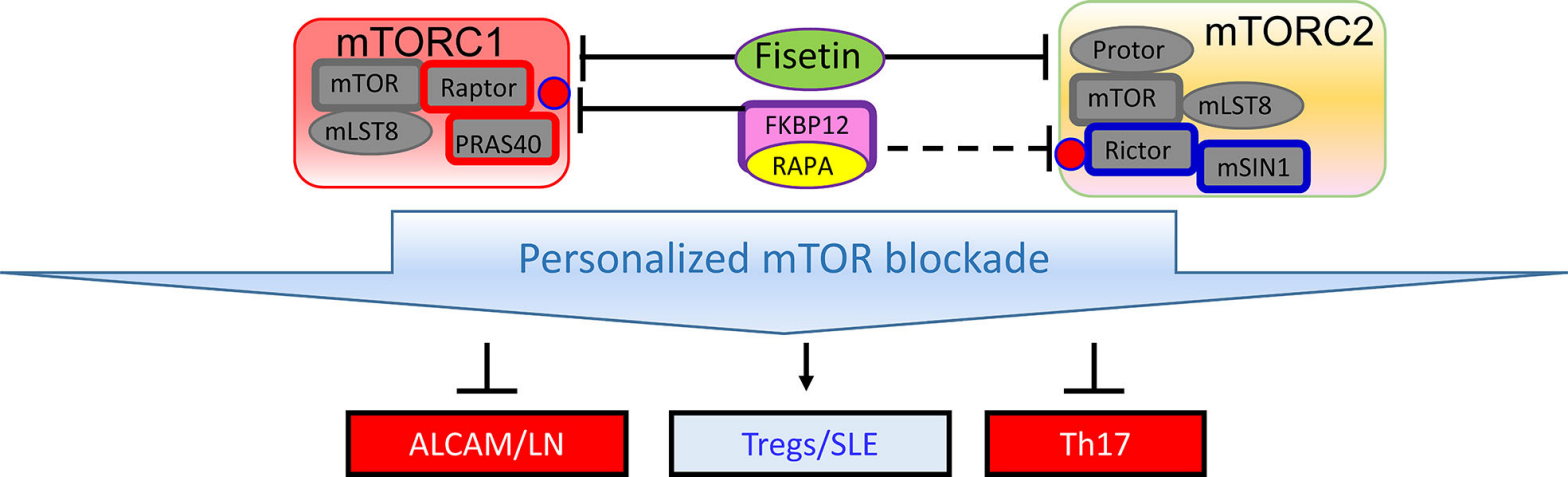
Schematic diagram of mTOR complexes 1 (mTORC1) and 2 (mTORC2) in SLE. Similar to rapamycin, fisetin inhibits both mTOR complexes (34), which may be involved in expanding Tregs (35) and thus improving depression and other organ involvement in SLE (10, 35).

In conclusion, while a single marker may not adequately capture all the finesses of LN, SLE-CVD, NPSLE or other subcategories of SLE, it is conceivable that the creation of easily accessible screening assays measuring one or more factors will provide fast and reliable information about the development of organ-specific symptoms, the onset of flares, and the prediction of different therapeutic intervention among diverse SLE patients. Before we can reach such goal, it is however important that new markers are tested across different patient groups. For example, it will be of interest to know if S100A8, PF4 and (s)VCAM-1 are similarly upregulated in SLE patients with NPSLE or CVD, or if this phenotype is specific for LN. Future studies are clearly warranted to substantiate the importance of these biomarkers for the diagnosis and treatment of SLE.

## Author contributions

AP conceived and wrote the paper. All authors contributed to the article and approved the submitted version.

## Funding

This work was supported in part by grants AI072648 (AP), AI122176 (AP), and AR076092 (AP), and R01 AI118774 (TJ) from the National Institutes of Health, the Phillips Lupus and Autoimmunity Center of Excellence (AP), and the Central New York Community Foundation (AP).

## Conflict of interest

The authors declare that the research was conducted in the absence of any commercial or financial relationships that could be construed as a potential conflict of interest.

## Publisher’s note

All claims expressed in this article are solely those of the authors and do not necessarily represent those of their affiliated organizations, or those of the publisher, the editors and the reviewers. Any product that may be evaluated in this article, or claim that may be made by its manufacturer, is not guaranteed or endorsed by the publisher.
